# Supplementary material for: Quality, productivity, and economic implications of exoskeletons for occupational use: A systematic review
Source: PLoS One. 2023 Jun 27;18(6):e0287742. doi: 10.1371/journal.pone.0287742 (PMC10298758; doi:10.1371/journal.pone.0287742)
Supplement: S1 Appendix — (DOCX) [file pone.0287742.s002.docx]

Screening Tool

Q1. Is this article a review paper? If yes, EXCLUDE.

Q2. Is this article written in language other than English? If yes, EXCLUDE.

Q3. Does this article focus on the use of exoskeleton? If yes, INCLUDE.

Q4. Does this article use exoskeletons in the context of rehabilitation? If yes, EXCLUDE.

Q5. Does this article measure quality metrics (e.g., electromyography, range of motion, etc.) without relating them to productivity and/or quality? If yes, EXCLUDE.

Q6. Does this article relate the use of exoskeletons to a work setting or occupational tasks? If yes, INCLUDE.

Q7. Does this article relate the use of exoskeletons to quality, productivity, or economic impacts? If yes, INCLUDE.
