# Supplementary material for: Quality, productivity, and economic implications of exoskeletons for occupational use: A systematic review
Source: PLoS One. 2023 Jun 27;18(6):e0287742. doi: 10.1371/journal.pone.0287742 (PMC10298758; doi:10.1371/journal.pone.0287742)
Supplement: Attachment — Submitted filename: Response to Reviewers.docx [file pone.0287742.s004.docx]

**We would like to thank both reviewers for their comments and the academic editor for the opportunity to improve this manuscript. We are pleased that reviewers found this manuscript to be well written and satisfactory. Please see our responses to each comment below.**

Reviewer #1: In this manuscript authors analyzed the effects of exoskeleton use on Quality and Productivity. For this purpose 15 critical studies were selected and reviewed for the factors/measures of endurance time, task completion time, number of errors, number of task cycles completed etc. Following are the concerns and suggestion to improve the manuscript

Thank you for your constructive feedback. We believe the following modifications significantly improved the manuscript.

1. As per my understanding, the author's intension was to measure the quality and productivity of produced, developed and available exoskeleton in and out of lab applications. For this purpose keywords selected were not focused and had deficiencies, like human power augmentation, HCI based exoskeleton, BCI based exoskeletons, neurorobotics etc. These keywords may provide other critical studies for the mentioned topic.

Thank you for your comment. The intention of this systematic review was not to evaluate the quality and productivity of exoskeletons themselves. Instead, the purpose of this systematic review was to synthesize the current state of knowledge on the quality and productivity of workers and their work while wearing exoskeletons, as well as the economic implications of exoskeletons for occupational use. For example, we are interested in how the usage of exoskeletons could impact construction or assembly line workers’ quality and productivity of their work while wearing exoskeletons.

In order to avoid possible confusion, we have added some clarification in the Introduction (lines 79-81).

2. The Introduction section is very brief and lacks a comprehensive review including introduction of exoskeleton, their types and applications. This will help to better contextualize your study and highlight the significance of your research question.

Thank you for your feedback. We have added additional information to further clarify the types and applications of exoskeletons (lines 42-43). We have also cited several studies to help readers obtain more in-depth information regarding different types of exoskeletons (lines 48-51).

3. Table 3 provides the good insights of the previous studies, however a critical review selected factors/measures is missing to answer the research question. Enhance this section of the manuscript, I recommend to categorize this section with respect to the selected factors/measures and impact on type of exoskeleton. This will help to highlight the strengths and weaknesses of previous research, and provide a clearer picture of how your study contributes to the field.

Thank you for your feedback. The purpose of our systematic review was to synthesize the current state of knowledge on the quality and productivity of workers and their work while wearing exoskeletons, as well as the economic implications of exoskeletons for occupational use. Performing a critical review of the measures on quality and productivity of exoskeleton products was beyond the scope of our systematic review. We understand that this might be a source of confusion and therefore, in response to your comment, we further clarified our research objective in the Introduction (lines 79-81).

4. With respect to quality and productivity, commercial exoskeletons were not reviewed and ignored. In order to complete the comprehensive review, commercially available exoskeletons should also be included in the study, this may enhance the contribution of this study and helps to suggest conclusive recommendations.

Thank you for your comment. We did not exclude commercial exoskeletons in our search strategy. “Exoskeleton” is one of the terms used in our literature search. Some of the papers that were retained for our review based on our systematic search and screening process, assessed the commercial exoskeletons designed for occupational application such as Laevo, HAL, BackX, and EksoWorks, and some assessed their own proprietary exoskeletons (Table 3).

5. The manuscript does include a brief discussion section, however recommendations are ignored or mixed with discussions. Comprehensive and conclusive findings and recommendations are missing, it is suggested to review all these sections and include additional headings to support the findings and arguments.

Thank you for your feedback. In response to your comment, we have provided recommendations for future studies in the new Section 4.1 to better guide the reader.

We clarified our conclusion in Section 4.3. Our systematic review provided mixed evidence regarding the effectiveness of exoskeleton based on quality and productivity measures. We also observed an absence of evidence on the economic impacts (e.g., cost, return on investment) of exoskeletons for occupational use. When selecting and adopting exoskeletons, task characteristics (e.g., required movements, workspace) may need to be considered to achieve the most effective outcomes for quality and productivity of workers and their work. More empirical studies are needed to improve our understanding on quality, productivity, and economic impacts of exoskeletons, including studies that consider sex and gender as well as studies that take place in actual work environments.

Reviewer #2: Overall, the presented paper is satisfactory. However, it is crucial to delve deeper into the factors that influence the quality and productivity outcome measures when utilizing an exoskeleton for occupational tasks. Additionally, it is recommended to review the excluded criteria in the screening step. Please revise them accordingly.

Thank you for your comment. We appreciate that you found this paper satisfactory.

In this systematic review, our objective was to synthesize the state of knowledge on the quality and productivity of workers and their work while wearing exoskeletons, as well as the economic implications of exoskeletons for occupational use. Assessing the factors that influence the quality and productivity outcome measures was beyond the scope of our systematic review. In response to your comment, we have clarified our inclusion and exclusion criteria in Section 2.2.

Kindly attach the checklist of questions pertaining to the inclusion criteria of the papers for reference. This will help ensure the selection process is comprehensive and accurate.

Thank you for your feedback. We have attached our screening tool in S1 Appendix. It includes the checklist of questions pertaining to the inclusion criteria.

Lastly, to enhance the validity of the results, it is advisable to increase the number of papers included in the review. This will provide a more robust foundation for validating the findings.

Thank you for your comment. We followed the systematic review guidelines and PRISMA checklist to obtain the most relevant research articles pertaining to our research objective. Our search strategy included six large databases (i.e., PubMed, Medline, Embase, Business Source Complete, IEEE Xplore, and Scopus) and we identified 6,722 articles in our initial search. We had to exclude 670 duplicates as well as 6,037 articles because they did not meet the inclusion criteria.
